# Porcine Circovirus Type 3: Diagnostics, Genotyping, and Challenges in Vaccine Development

**DOI:** 10.1155/2023/8858447

**Published:** 2023-09-28

**Authors:** Stella Pranoto, Hsing-Chieh Wu, Chun-Yen Chu

**Affiliations:** ^1^International Degree Program in Animal Vaccine Technology, International College, National Pingtung University of Science and Technology, Pingtung, Taiwan; ^2^Graduate Institute of Animal Vaccine Technology, College of Veterinary Medicine, National Pingtung University of Science and Technology, Pingtung, Taiwan

## Abstract

Porcine circovirus (PCV) comprises four distinct species, namely, PCV1–4, with considerable differences between them, resulting in limited crossprotection. PCV2 inflicts substantial economic losses on the swine industry. PCV3 was found to have been circulating before its discovery in 2015. PCV3 is suspected of having a comparable impact to PCV2; however, its characterization studies are still underway. The symptoms of PCV3 infection are similar to those of PCV2 infection. However, PCV2 and PCV3 share a maximum amino acid identity of only 37%, which partially explains the inadequacy of the PCV2 vaccine in protecting against PCV3 infection. Swift measures must be taken to control PCV3, including vaccine development, as it also poses a threat to swine populations. This review synthesizes the recent findings on PCV3 in comparison with PCV2 and highlights the prevailing challenges encountered in vaccine development. Various strategies and innovative approaches to producing PCV3 vaccines, such as using RNA particle technology and baculovirus vectors, are also discussed. Addressing research gaps in diagnostic methods, viral isolation, and vaccine development is crucial to controlling this virus, which poses a threat to swine populations.

## 1. Introduction

The discovery of porcine circovirus (PCV) dates back to 1974 when a German research team found a picornavirus-like contaminant in porcine kidney (PK-15) cell culture, which was later designated as PCV type 1 (PCV1). PCVs belong to the *Circoviridae* and the genus *Circovirus*. They are considered one of the smallest viruses, with a diameter of approximately 17–20.7 nm [1], and possess single-stranded circular DNA. Another member of this genus is the beak and feather disease virus [2, 3].

To date, four species of PCV have been identified: PCV1, PCV2, PCV3, and PCV4. Notably, although the names are the same, the types are species names, not strains or variants; thus, there are prominent differences among them, resulting in limited crossprotection. PCV1, commonly found in pigs, is widely accepted to be nonpathogenic. By contrast, PCV2 causes significant economic losses to the swine industry. The newly emerged PCV3 was suspected of having an impact similar to that of PCV2; however, many characterization studies are still ongoing. Moreover, even less information is available on recently identified PCV4 [4].

PCV2 has been identified as the etiological agent of postweaning multisystemic wasting syndrome (PMWS). It was first described in Canada in 1995. Since then, PCV2 has been extensively studied, and even a vaccine has been commercialized to control this virus. However, the viral spread continues and is endemic worldwide [4]. PMWS was one of clinical manifestation of PCV-associated disease (PCVAD). Beside PMWS, PCVAD also includes reproductive failure, enteritis, respiratory disorders, porcine dermatitis and necropathy syndrome (PDNS), and central nervous system (CNS) disorder as the possible clinical signs [5].

Although its pathogenesis remains unclear, PCV2 primarily targets lymphoid tissues, leading to lymphatic depletion, enlarged lymph nodes, and further immunosuppression in pigs. Pigs are prone to coinfections that amplify clinical signs. Coinfections with porcine parvovirus, porcine reproductive and respiratory syndrome virus, and *Mycoplasma hyopneumoniae* are often found [5].

Thus, the economic impact of PCV2 is significant. The fact that it was subclinical made it difficult to predict the swine industry production because the symptoms mostly developed at later stage of the swine growth. Morbidity varies from 4% to 60%, whereas mortality is approximately 4%–20% [6]. An assessment calculated that in 2008, the loss in England due to PCV2 was £52.6 million [7]. Worldwide losses were not mentioned before the introduction of the vaccine.

In 2015, PCV3 was first identified in North Carolina, United States, in sows with PDNS-like symptoms, reproductive failure, cardiac pathology, and multisystemic inflammation. Shortly thereafter, it was also found on all continents worldwide, reporting countries including China, South Korea, Japan, Thailand, Vietnam, India, Poland, Germany, Denmark, Spain, Italy, Mozambique, Nigeria, Tanzania, Brazil, Mexico, Colombia, Argentina, Chile, and Canada [5, 8–15]. PCV3 shared a maximum amino acid identity of 37% with PCV2; this partially explains why the PCV2 vaccine does not protect against PCV3 infection. Rapid action should be taken to control PCV3 considering its potential economic impact. This review aims to summarize updates on newly discovered PCV3, its relationship with PCV2, and the current challenges in vaccine development.

## 2. Subclinical and Clinical Signs

Both PCV2 and PCV3 were present in subclinical or clinical infections. Subclinical infection is the most common occurrence of PCVAD, accounting for up to 70% of the cases. This usually happens from a single infection of PCV, without the presence of other pathogens. In the case of PCV2 subclinical infections, there are no more obvious clinical signs than decreased average daily gain [6]. Meanwhile, the clinical manifestations of PCVAD often depend on the coinfections. Therefore, it can be varied, including PMWS, reproductive, respiratory, enteric, PDNS, and in some cases, CNS syndromes. It's hard to distinguish PCV2 and PCV3 infections just based on the symptoms because some reports on PCV3 mentioned similar signs as PCV2, especially respiratory, reproductive failure, and multisystemic inflammation [5, 10, 16]. Coinfection of these two viruses was also discovered at a rate of 16.7%–18.6% [17, 18]. However, Visuthsak et al. [18] found no correlation between PCV2–PCV3 coinfection and clinical manifestation. More studies are warranted to prove the onset of PCV3-associated disease.

Several studies also aimed to reproduce PCV3-induced disease to understand its pathogenicity. Some reported that PCV3 mostly also produced subclinical infection [19, 20]. Inoculation of CD/CD pigs with PCV3-positive tissue homogenate did not show any clinical signs, even the average daily weight gain was comparable to the control group. However, PCV3 infection was confirmed by quantitative polymerase chain reaction (qPCR) in serum (3–42 dpi) and nasal secretion (3–28 dpi) and by in situ hybridization (ISH) in multiple organs, such as brain, heart, liver, kidney, and small intestine. Histological evaluation also confirmed the presence of the virus, although only at the late stage of infection (42 dpi) [20]. From 33 PCV2-vaccinated farms in Thailand, PCV3 DNA was found in all pigs of different production stages, either with (22.45% positive rate) or without (47.70% positive rate) clinical symptoms, although the viral load detected in symptomatic cases was slightly higher than the asymptomatic cases [18].

The most common symptom of PCV infections is multisystemic inflammation. Histological lesions found were mostly characterized by myocarditis and systemic perivasculitis [16, 19, 20]. A study reproduced PDNS-like disease by inoculating infectious PCV3 DNA clones into SPF piglets, resulting in the reduction of lymphocyte proliferation in the peripheral blood. The lymph nodes were seen two to three times larger and firmer with hyperplasia compared to the healthy ones. A hyperemic liver was also observed, along with gray-white nodules and necrosis [5].

Reproductive failure was the first symptom recognized in the first case of PCV3 in North Carolina, USA. The farm experienced a 1.19-fold increase in abortions per litter compared with the average rate. The sow mortality rate increased by 10.2%, and the conception rate decreased by 0.6%. Palinski et al. [21] detected mummified fetuses of various gestational ages with symptoms of PCVAD, as described in PCV2-associated cases. Rather than PCV2, they discovered a novel type of PCV (PCV3) using metagenomic sequencing [21]. In Spain, PCV3 DNA was found in 18/53 cases of reproductive failure. Coinfection with PRRSV, PCV2, and PPV1 was ruled out in 16 of these cases. Most fetal deaths occur during the last third of gestation. The crown-to-rump length of stillborn piglets is 6–32 cm, suggesting intrauterine transmission [22]. Abortion, stillbirth, and repeat breeding of newborn piglets with swollen legs have been reported in India. Forty percent of piglets die within the first week of life, whereas the remaining 60% die within 6 months and show poor performance [10]. These reports highlight the potential of PCV3 as a causative agent of swine reproductive diseases.

Respiratory diseases, mainly characterized by dyspnea, are similar to PCV2-associated diseases. Nasal discharge and coughing were observed, most likely caused by interstitial pneumonia, with variable degrees of congestion, edema, and emphysema in the lungs [10]. Postmortem examination of the reproduced diseased piglets showed rubbery lungs with a diffuse mottled tan-red appearance and severe multifocal dark purple-to-red consolidation [5].

Another obvious clinical sign that is easily detected is PDNS. They appear as multifocal papules and macules in sows, facial edema in newborn piglets, and proliferative dermatitis on the dorsal surface of the pinna [5, 10, 21].

The CNS may be affected by PCV3. This virus has been detected in neonatal piglets with congenital tremors (CT). Coinfections were found, notably with atypical porcine pestivirus, which may be related to this CT symptom. However, the two PCV3-positive samples were negative for atypical porcine pestivirus, indicating that PCV3 contributes to CT. The mortality of CT-affected piglets is 100% because of their inability to stand or walk [23]. Thus, PCV3 may cause PMWS; reproductive, respiratory, and enteric diseases; PDNS; and CNS disorders. Further studies of its pathogenesis are required to understand the disease onset.

PCV3 spreads through horizontal and vertical transmission, similar to PCV2 transmission [24]. PCV3 has been detected in various body fluids such as saliva, nasal fluids, urine, and feces, suggesting horizontal transmission through direct contact. Vertical transmission can occur through infected semen or colostrum of sows lacking maternal antibodies against PCV3 [14]. Reports of reproductive failures, such as massive mortality of neonatal piglets and variable crown-to-rump lengths of mummified fetuses, suggest intrauterine transmission routes [23, 25, 26].

## 3. Diagnostic Methods

As PCVAD is mainly asymptomatic in the early stages of infection, various approaches should be used for its diagnosis. The same case happens with PCV3, as it could produce subclinical infection. The role of PCV3 as the causative agent was still debated because of the lack of legitimate proof. The presence of genomic material in the pig does not correlate with the disease. To establish a new disease-causing agent, at least these criteria must have been mentioned: (1) consistent clinical symptoms, (2) histological lesions, and (3) the presence of the agent in such lesions [22]. In addition to clinical observation and polymerase chain reaction (PCR), histological method is required to confirm the disease.

PCR is the easiest way and widely used to diagnose infectious diseases. The detection of PCV3 in the tissue and blood samples supports the diagnosis of the disease caused by this newly emerging virus. In the first case of a PCV3 outbreak, the virus was detected in fetal tissue homogenates, serum, and lesioned tissues of sows [21]. The target gene for PCV3 is the open reading frame 2 (ORF2), which encodes a capsid protein.

Various organ samples have been tested using PCR in other studies. Most research stated that the virus has mainly been detected in the lung tissue of aborted fetuses or stillborn piglets [10, 16, 27, 28]. Serum and nasal swabs from the affected sows or other piglets aged 3–21 weeks old also tested positive for PCV3. However, the genome copies were much lower than that of stillborn piglets' tissues [10, 29].

PCV3 genome was also found in other fetal organ samples, such as the heart, liver, kidney, spleen, naval cord, lymph node, tonsil, and brain [10, 16, 23], but in different levels of genome copies. Bera et al. [10] reported that the highest genome copy number of PCV3 was detected in the heart and naval cord of the stillborn piglets. Chen et al. [23] observed the largest number of viral genomes in the heart and brain. These findings may correlate with the clinical manifestations in each case. However, the primary replication site of PCV3 remains unknown. Whether this result correlates with the route of infection or other factors should be further examined [26].

In addition to PCR, immunohistochemistry is a simple yet necessary method for diagnosing PCV3, particularly in subclinical cases. The most commonly sampled organs are lungs, liver, kidneys, spleen, lymph nodes, heart, and sometimes small intestine or naval cord of stillborn piglets [5, 10, 21]. A positive result was observed in microscopic lesions showing lymphocyte infiltration into these organs, followed by dysplasia and necrosis, suggesting a disorder in the piglet immune system, thus enhancing the possibility of coinfections with other diseases. With the presence of coinfections, more obvious clinical symptoms usually appear, and morbidity and mortality increase [5, 10]. Lesions mostly found in fetuses, stillborn, or weak-born piglets were myocarditis, periarteritis, and/or encephalitis. Meanwhile, in weaned piglets, myocarditis, systemic periarteritis, and dermatopathy leading to necrotizing vasculitis were often present [22].

Serological detection methods, such as enzyme-linked immunosorbent assay (ELISA), immunofluorescence assay, and immunoperoxidase monolayer assay, are less suitable for PCV3 diagnosis. In the case of PCV2, owing to ubiquitous exposure to the virus worldwide, many pigs develop anti-PCV2 antibodies because of a previous infection that is most likely subclinical [30]. However, PCV2 ELISA kits are commercially available mainly for vaccination evaluation. ELISA detection of PCV3 using recombinant capsid protein is also possible, although it is less common, partly because of the similar tendency of subclinical infection, and the development of the kit itself is still ongoing.

Isolation is an essential step in the detection and study of viruses. PCV2 can replicate in PK-15 cells, Vero cells, and other porcine-derived cell lines [30], and several attempts have been made to isolate PCV3 (Table 1). By contrast, PCV3 isolation attempts using PK-15 and ST cell lines were unsuccessful. Neither ISH nor qPCR showed positive results for viral replication, even after three passages [10, 21, 31]. This raises the question of what distinguishes PCV2 from PCV3. One hypothetical answer could be that virus entry may require different receptor conformations, considering that PCV2 and PCV3 only share 36%–37% similarity.

The first successful isolation was performed using primary PK cells from a 21-day-old crossbred piglet. Eight passages were performed and monitored by ISH, transmission electron microscopy (TEM), and qPCR. PCV3 was isolated from the inguinal lymph node of a clinically healthy pig aged 91 days old with markedly low genomic copies (2.33 × 10^4^ genomic copies/mL). Although no cytopathic effects were observed, ISH confirmed that the virus was predominantly present in the cytoplasm. TEM at 72 hr postinoculation revealed large irregular inclusion bodies with a virus-like particle (VLP) arrangement, which were suspected to be PCV3. Additionally, qPCR revealed that the genomic copies of PCV3 increased approximately 10-fold until the sixth passage but remained stationary until the eighth passage. Despite successful isolation, homologous and highly permissive primary PK cells for PCV3 infection require further investigation [31].

Another successful method of reproducing PCV3 is the generation of an infectious clone. Jiang et al. [5] cloned the full PCV3 genome into pBlueScript SK. The infectious clones were rescued using PK15 after 15 passages. The clone was found to be quite stable, as no mutations were detected after several passages. Immunofluorescence staining revealed that PCV3 replicated predominantly in the cytoplasm but some replicated in the nucleus. The titer at passage 15 was 10^6.53^ TCID_50_/mL. This infectious clone successfully induced a PDNS-like disease in SPF piglets via the intranasal route [5].

## 4. Genotyping

Similar to other *Circovirus* genomes, PCV3 has circular single-stranded DNA around 1,999–2,003 bases long, slightly longer than PCV2, which only has 1,766–1,777 bases. Several studies have reported three ORFs with structures similar to those of PCV2. ORF1 encodes replicase, an enzyme of 296–297 amino acids. In an orientation opposite ORF1, ORF2 translates to the only structural capsid protein (214 amino acids). However, ORF3, which overlaps with ORF1, has an unknown function. The start codon itself remains unclear; thus, it may result in a 231-aa or 177-aa-protein [31, 32].

Among all the ORFs, ORF1 (replicase) is the most conserved; therefore, it is often used to analyze the virus origin. ORF1 of PCV3 shares 54% amino acid similarity with bat circovirus; therefore, it is suspected that PCV3 was originally derived from bat circovirus clade 1, not another type of PCV. In comparison, the similarity to PCV2 is only 36%–37% [21]. PCV1 and PCV2 are closely related to the bat circovirus clade 2 [33].

PCV3 is believed to be circulating in either domestic pigs or wild boars long before its first identification. Its most common ancestor was believed to have originated around 1966 [14]. *Circovirus*, in general, can infect a wide range of hosts. Cross-species infections with PCV3 have also been reported in other mammals. Cattle in Shandong Province, China, were infected with PCV3 at a prevalence of 34.7% without any clinical symptoms. Analysis of the capsid sequence revealed genetic markers of PCV3 across different species. In cattle, Y124 is present instead of D124 (dogs or pig-born). In dogs in Hunan Province, China, a prevalence of 10% was detected, and a specific V206A mutation was found [34, 35]. These genetic markers are important for characterizing the virus based on host species and understanding the transmission route. In addition to cattle and dogs, PCV3 has been reported to infect laboratory mice, BALB/c, and ICR, making it possible to study this virus using mice as a model [36]. The study of the PCV3 host range should be continued, as it provides information on the possible reservoir of the virus.

Until 2018, sequence identities among sequences reported in GenBank were considered as high (>97%) for either partial or complete PCV3 sequences [14]. Although there are few genetic variations in PCV3, genotyping was proposed by Li et al. [33]. PCV3 was suspected of diversifying between 2013 and 2014, right before the outbreak in North Carolina. Two stable clades have been proposed: PCV3a and PCV3b. PCV3a is further divided into PCV3a-1 and PCV3a-2. Some random strains that appeared once and did not branch were classified as PCV3a-IM (intermediate strains). This classification was calculated using neighbor-joining, maximum-likelihood, or Markov chain Monte Carlo methods on the complete coding sequences (ORF1 + ORF2); this is significantly different from the PCV2 classification, which uses ORF2 only. By contrast, the tree constructed using ORF2 of PCV3 did not show a clear cluster and was inconsistent when reference strains were added [33].

Until now, there is no universally accepted classification system for PCV3. Other published studies have used either ORF2 only or the whole genome to classify PCV3, therefore it results in a different grouping. Classification using the whole genome often resulted in two distinct groups [28, 32, 34]. Yue et al. [28] constructed a phylogenetic tree based on the amino acid alignment of ORF2 and divided PCV3 into three groups: 3a, 3b, and 3c. In another case, Fux et al. [32] determined group a1, a2, b1, and b2 based on ORF2 DNA sequence alignment. However, the group naming in different study is incomparable with each other. For example, a USA isolate (accession number KX778720) was classified as group 3a-1 [33], 3a [28], 3b [34], and b1 [32]. More sequences seem to be required to establish a universal standard for PCV3 subspecies classification criteria.

Specific amino acid mutations were found in each group (Table 2), which can be used as markers. Out of 94 ORF2 sequences from North and South America, several polymorphisms were detected. Seven of them were located outside of the virion model: amino acids position 56, 75, 77, 98, 101, 150, and 155, which are supposed to be accessible to immune cells [9]. Since the change of charge or polarity could cause different affinity to the host immune system, as demonstrated in PCV2 cases, the correlation between these motifs and antigenicity or other biological properties remains to be a future work [28, 32–34, 37]. Klaumann et al. [14] reported that the PCV3 genome has been relatively stable since the first outbreak in 2015. By contrast, evolutionary dynamics study showed that PCV3 has a relatively high reproductive number value, which means that it has the potential for continuous outbreak and cross-species transmission [33]. Considering its potential implications, the immediate implementation of effective preventative measures is necessary.

The correlation between clade and geographical distribution was weak. All clades were present in the reported countries, including the USA, Brazil, the UK, Spain, Poland, Germany, Italy, Denmark, China, Thailand, Malaysia, Vietnam, and South Korea [16, 31, 32, 38]. Based on the association index and parsimony score of the sequences from 2015 to 2017, the monophyletic clade was significant only in China [33].

## 5. Virus Structure

The structure of PCV3 is similar to that of other circoviruses and resembles a *T* = 1 icosahedral virion. Each virion consists of 60 subunits of the capsid protein, the only structural protein. VLP of PCV3 was successfully generated, resulting in 7.5–10 nm size particles, almost twice as small as the isolated virus reported by Oh and Chae [31] and Bi et al. [39] (17 nm). VLP helps understand the structure and potential epitopes of PCV3, as isolation of this virus is still in progress.

**Figure 1:**
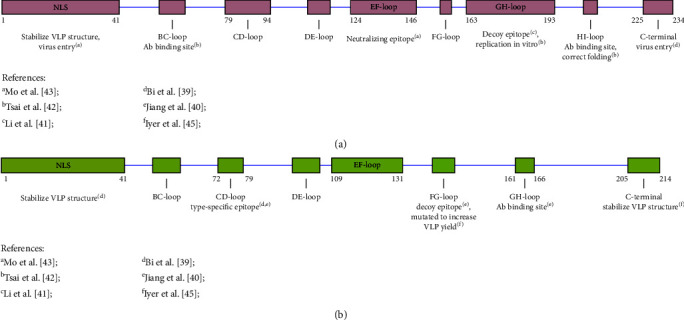
Epitope mapping of PCV2 (a) and PCV3 (b) and its possible roles.

Several studies have attempted to map and identify PCV3 epitopes, representing an important step in vaccine development. Each secondary capsid structure consists of one *α*-helix nuclear localization signal (NLS) region and *β*-sheets as the core is connected by seven surface loops (Figure 1). On the other hand, in PCV2 capsid protein, there are four known antibody recognition regions, aa 51–84, aa 113–139, aa 161–207, and aa 228–233, which contribute to strain specificity. Amino acid position 190–191, 206, and 210 play a role in PCV2 replication in vitro [42]. Regions *β*3 and *β*6 are highly conserved between all types of PCVs; thus they are hypothesized to have a vital role in virus survival, possibly in particle assembly, host entrance, or viral replication. Meanwhile, the seven loops were predicted as linear B-epitopes using BepiPred web-based software. To test this prediction, Jiang et al. [40] identified three linear epitopes, aa 57–61, aa 140–146, and aa 161–166, which bind to PCV-specific mAb and PCV3-positive serum (Figure 1(b)). Interestingly, aa 140–146, which had the highest reactivity, was similar to the PCV2 decoy epitope (Figure 1(a)) and conserved among all types of PCVs. As the most immunodominant epitope is predicted to be non-neutralizing, it could be a viral trick to escape the host immune response [39–41].

Cryo-EM structural comparison revealed some notable differences between the PCV3 and PCV2 virions, pinpointing the specific epitope of PCV3; they specifically differed in the CD loop (aa 72–79), EF loop (aa 109–131), and NLS regions. The CD loop of PCV3 is two times shorter than that of PCV2, and replacement with the CD loop of PCV2 compromised binding reactivity to PCV3-specific mAbs. Based on this study, the CD loop is a PCV3 type-specific epitope (Figure 1(b)). However, the neutralization capacity of the resulting antibodies requires further evaluation. By contrast, the PCV2 type-specific neutralizing antibody is located in the EF-loop (aa 128–143) (Figure 1(a)) [39, 43].

The unique folding of the NLS region in PCV3 creates protruding regions on the VLP compared with that in PCV2, which is flatter. It is difficult to use the NLS region for recombinant protein expression because it contains arginine-rich residues rarely produced by *Escherichia coli*. Some studies have preferred to express N-truncated capsid proteins, which could increase the yield, but unfortunately, failed to generate VLP [44]. In this case, the NLS of PCV3 may have the same stabilizing function as that of PCV2. In addition to stabilizing the structure, the NLS in PCV2 functions as a cell-penetrating peptide to facilitate the entry of the virus [39, 43]. This NLS variation in PCV3 may contribute to its incompatibility with PK-15 cell lines.

## 6. Recent Development of PCV3 Vaccines

Because of the importance of PCV3 as the proposed pathogen and the lack of crossprotection between PCV2 and PCV3, it is crucial to develop novel vaccines against PCV3. Recent development of PCV3 vaccines is summarized in Table 3. Referring to PCV2 successful recombinant vaccine, we can use a similar strategy to develop a PCV3 vaccine. However, to date, no vaccine against PCV3 is available in the market. The only preventive option is offered by Merck Animal Health is the autogenous PCV3 vaccine using RNA particle technology under the Sequivity® platform, but this vaccine needs 8–12 weeks to custom [47]. The biggest challenge in developing a PCV3 vaccine supposedly lies in proper efficacy testing for unculturable viruses.

PCV2 commercial vaccines are efficacious in controlling clinical symptoms and economic losses. It started with inactivated PCV2, an inactivated baculovirus vector containing the PCV2 capsid protein, inactivated chimera PCV1–PCV2, and the most popular subunit vaccine consisting of the PCV2 capsid protein (ORF2). This suggests that capsid protein alone protects domestic pig populations against PCV2. Subunit vaccines are currently more desirable because of their safety, ease of modification, and choice of recombinant system production. Moreover, the capsid proteins of PCV2 tend to form VLP, enhancing their recognition by the immune system. However, the introduction of the PCV2 vaccine subtype 2a seems to have affected viral evolution and generated other genotypes (2b, 2d) [48].

In 2020, Boehringer Ingelheim filed a patent for producing the PCV3 vaccine, which is also a VLP from the capsid protein (ORF2) produced by the recombinant baculovirus vector. The vaccine was adjuvanted with Montanide ISA27VG or 20% Carbopol. This vaccine may be commercialized in combination with other antigens such as PCV2, *M. hyopneumoniae*, pseudorabies, swine influenza, classical swine fever, African swine fever, *Actinobacillus pleuropneumoniae*, swine *E. coli*, porcine parvovirus, and *Pasteurella multocida* [45].

Some modifications of ORF2 are stated in the patent file. These mutations mostly mimic PCV2 ORF2 and aim to stabilize VLP and increase yield during production. The original sequence of the PCV3 full-length capsid protein formed only 20% of the assembled VLP, while the remaining 80% aggregated into the insoluble fraction owing to its highly hydrophobic nature. The first modification was a mutation in the FG-loop, which increased VLP yield from 20% to at least 50%. Another mutation was an extension of the C-terminus. As PCV3 capsid C-terminus is short, hydrophobic, and lacks nucleic acid in VLP, it would lead to being buried and unstable. This extension can be performed using either random amino acids, C-terminal amino acids from PCV2 ORF2, or the next stop codon in the viral sequence [44, 45].

Regarding challenge viruses for efficacy testing, the options are to use a PCV3-positive tissue homogenate, PCV3 whole virus, or an infectious clone. Tissue homogenates must be screened for extraneous agents using qPCR and sequencing. Quantification of the virus was performed using the genomic copy number of the viral load. Although this method has the limitation of misinterpreting genomic copies with the virus functional titer, it appears to be the simplest and most plausible method. Challenges with the whole virus or tissue homogenate were boosted by the administration of keyhole limpet hemocyanin emulsified in incomplete Freund's adjuvant as an immunostimulant. The results showed that tissue homogenates were more infectious than whole viruses [45].

An infectious clone can be considered another option for challenge material because its first rescue was able to induce PDNS-like symptoms. This infectious clone killed two of five 4-week-old piglets with specific pathological lesions in their organs but was not fatal in 8-week-old piglets. The PCV3 antigen was detected by immunohistochemical staining of various organs postinoculation [5]. By contrast, the BI patent claims that the infection of the rescued clone is subclinical. Nasal shedding was detectable by PCR but not in fecal samples. However, the data were the least representative as only two animals per group were included in the study [45].

Another report described a novel technique for constructing a PCV3 infectious clone without the use of an expression vector. This clone was transfected into 3D4/21 cells and was able to infect the myocardium and alveoli of Kunming mice as an alternative challenge model [49]. Further research is needed to generate highly infectious clones as challenge materials for vaccine efficacy testing, thus helping progress in the development of the PCV3 vaccine.

Peswani et al. [46] developed an innovative vaccine candidate by constructing a chimeric subunit vaccine of the PCV2d–PCV3 capsid joined by a GS linker. Both PCV sequences were N-truncated, and only PCV3 was C-truncated. This vaccine candidate was expressed in *E. coli* as a cheaper fermentation system for vaccine production. During fermentation, these two fragments are cleaved to form single subunits of PCV2d and PCV3-derived peptides. The PCV2d subunit is soluble. Although it does not form VLP, it induces the production of PCV2d-neutralizing antibodies in rabbits. Unfortunately, this study lacked an assay for PCV3 immunogenicity [46]. With some options for the challenge model, we hope that further research on PCV3 vaccine development will be conducted.

## 7. Conclusions

PCV3, a distinct type of PCV, was found to have been circulating before its discovery in 2015. PCV3 shows symptoms that are thought to be similar to those of PCV2. However, successful PCV2 vaccines do not provide sufficient crossprotection against PCV3. Research gaps need to be addressed regarding the pathogenesis, diagnostic methods, viral isolation, and vaccine development to control this virus, which has recently endangered swine populations.

## Figures and Tables

**Table 1:** PCV3 isolation attempts.

Sample	Cell	Results	Reference
Inguinal lymph node from clinically healthy 91-day-old pig (PCV3 2.33 × 10^4^ genomic copies/mL)	Primary PK cells (kidney of 21-day-old crossbred piglet)	CPE: negative ISH: virus was predominantly in cytoplasms TEM: at 72 hr postinfection (hpi) showed large irregular intracytoplasmic inclusion bodies w VLP arrangement (suspected as PCV3) qPCR: viral genomic copies continued to increase till p6 and plateaued until the end of the experiment at p8	[31]
	PK-15	ISH: negative	

Fetal tissue homogenates	ST	3x passages: CPE: negative qPCR: Ct increase (not adapting well)	[21]
	PK-15	IFA: negative	

Tissue homogenate: lung, heart, liver, kidney, spleen and naval cord from stillborn piglet, and blood and nasal swabs from affected sow	PK-15	qPCR: Ct increase	[10]

Infectious clone: full PCV3 genome was cloned into pBlueScript SK	PK-15	IFA: PCV3 replicated in the cytoplasm (majority) and nucleus (minority) P15 titer: 10^6.53^ TCID_50_/mL	[5]

Tissue homogenate: lung, heart, and liver	PK-15	qPCR: genomic copies per mL increased at 12 hpi (3.61 × 10^8^), peaked at 60 hpi (5.16 × 10^8^), and slightly declined at 72 hpi (4.23 × 10^8^) CPE: negative IFA: p6 showed strong intracytoplasmic and/or nuclear fluorescence ISH: intracytoplasmic signals detected Sequencing: stable until p9	[19]

**Table 2:** PCV3 group-specific amino acid mutations.

Group^a^	ORF1	ORF2				ORF3		
	aa122	aa24	aa27	aa77	aa150	aa1	aa4	aa227
3a-1	S	A	R	S	I	S	G	V
3a-2	S	A	R	T	L	S	G	V
3a-IM	S	A	K	S	I	F	D/G	V
3b	A	V	K	S	I	F	D	G

*Note*: ^a^Group naming based on Li et al. [33]. S, serine; A, alanine; V, valine; R, arginine; K, lysine; T, threonine; I, isoleucine; L, leucine; F, phenylalanine; G, glycine; D, aspartic acid.

**Table 3:** Development of PCV3 vaccine.

Vaccine type	Description	Production system	Reference
VLP	Modified capsid protein of PCV3 adjuvanted with Montanide ISA27VG or 20% carbopol	Baculovirus vector system	[45]
Subunit	Chimeric PCV2d–PCV3 truncated capsid joined by GS-linker	*E. coli*	[46]
mRNA	Autogenous vaccine needs 8–12 weeks to custom	Synthetic RNA particle technology	Merck AH sequivity [47]

## Data Availability

The data that support the findings of this study are available from the corresponding author upon reasonable request.
